# The near-complete genome sequence of vB_AgrS_573, a lytic *Agrobacterium tumefaciens* phage isolated from karst cave sediment

**DOI:** 10.1128/mra.00484-26

**Published:** 2026-09-17

**Authors:** Qingshan Wu, Zhengmin Li, Yuanfeng Yang, Zheng Fang, Lan Xiang, Qingbei Weng

**Affiliations:** 1 School of Life Sciences, Guizhou Normal University 12686, Guiyang, People's Republic of China; 2 Qiannan Normal University for Nationalities 56700, Duyun, People's Republic of China; Katholieke Universiteit Leuven, Leuven, Belgium

**Keywords:** *Agrobacterium tumefaciens*, bacteriophages, whole genome sequence, genome analysis, *Caudoviricetes*

## Abstract

Phage vB_AgrS_573, isolated from oligotrophic karst cave sediment, is a lytic phage that infects *Agrobacterium tumefaciens* GV3101. It belongs to the class *Caudoviricetes* and harbors a 55,316-bp linear double-stranded DNA genome with 91 predicted protein-coding genes and a GC content of 52.19%; no transfer RNA genes were identified.

## ANNOUNCEMENT

*Agrobacterium tumefaciens*, the causal agent of crown gall disease, poses a severe threat to global crop production (1). Bacteriophages are promising biocontrol agents for bacterial plant diseases (2). Phages originating from oligotrophic environments may display enhanced replication efficiency, possibly through virus-encoded auxiliary metabolic genes that modulate host metabolism to support viral replication (3, 4). This potential has motivated the exploration and isolation of phage resources from such habitats. Here, we report the isolation and near-complete genome sequencing of lytic phage vB_AgrS_573. The phage was recovered in October 2025 from oligotrophic karst cave sediment (106°37′58″ E, 26°23′01″ N) in Guiyang, Guizhou Province, China, using *A. tumefaciens* GV3101 as the host strain.

Phage isolation was performed using previously described protocols (5). Briefly, 10 g of sediment was suspended in 90 mL of SM buffer, shaken at 120 rpm at room temperature for 20 min, centrifuged at 6,000 × *g* for 10 min, and filtered through a 0.22 µm membrane. One hundred microliters of the filtrate was mixed with *A. tumefaciens* GV3101 and plated onto top agar, followed by incubation at 30°C. Phage purification was achieved via four successive single-plaque isolations using the double-layer agar assay. Phage morphology was observed by transmission electron microscopy as previously described (6).

Viral genomic DNA was extracted from purified phage lysates (approximately 10^8^ plaque-forming units per milliliter) using a Takara viral genomic DNA extraction kit (Takara, Japan) following the manufacturer’s instructions. For Illumina sequencing, libraries were constructed with the TruSeq DNA Sample Prep Kit (Illumina, San Diego, CA, USA) and sequenced in paired-end mode (2 × 150 bp) on an Illumina NovaSeq platform at Shanghai Personal Biotechnology Co., Ltd. (Shanghai, China). A total of 28,253,426 paired-end reads were quality-controlled and trimmed using Fastp v0.22.0 (7). Clean reads were *de novo* assembled with SPAdes v3.12.0 (8), yielding a single linear contig of 55,316 bp. The assembly was then polished using Pilon v1.18 (9). Protein-coding genes were predicted with GeneMarkS v4.32 (10). Functional annotation was conducted using BLASTp (11) and the National Center for Biotechnology Information (NCBI) Conserved Domain Database (12). Genome mapping and visualization were carried out on the Proksee Server (https://proksee.ca/). Antibiotic resistance genes and virulence factors were predicted by homology searches against the Comprehensive Antibiotic Resistance Database (https://card.mcmaster.ca/analyze/rgi) and the Virulence Factor Database (http://www.mgc.ac.cn/VFs/). The genome-wide average nucleotide identity between phage vB_AgrS_573 and the closely related phages was calculated and plotted by Virus Intergenomic Distance Calculator (13).

**Fig 1:**
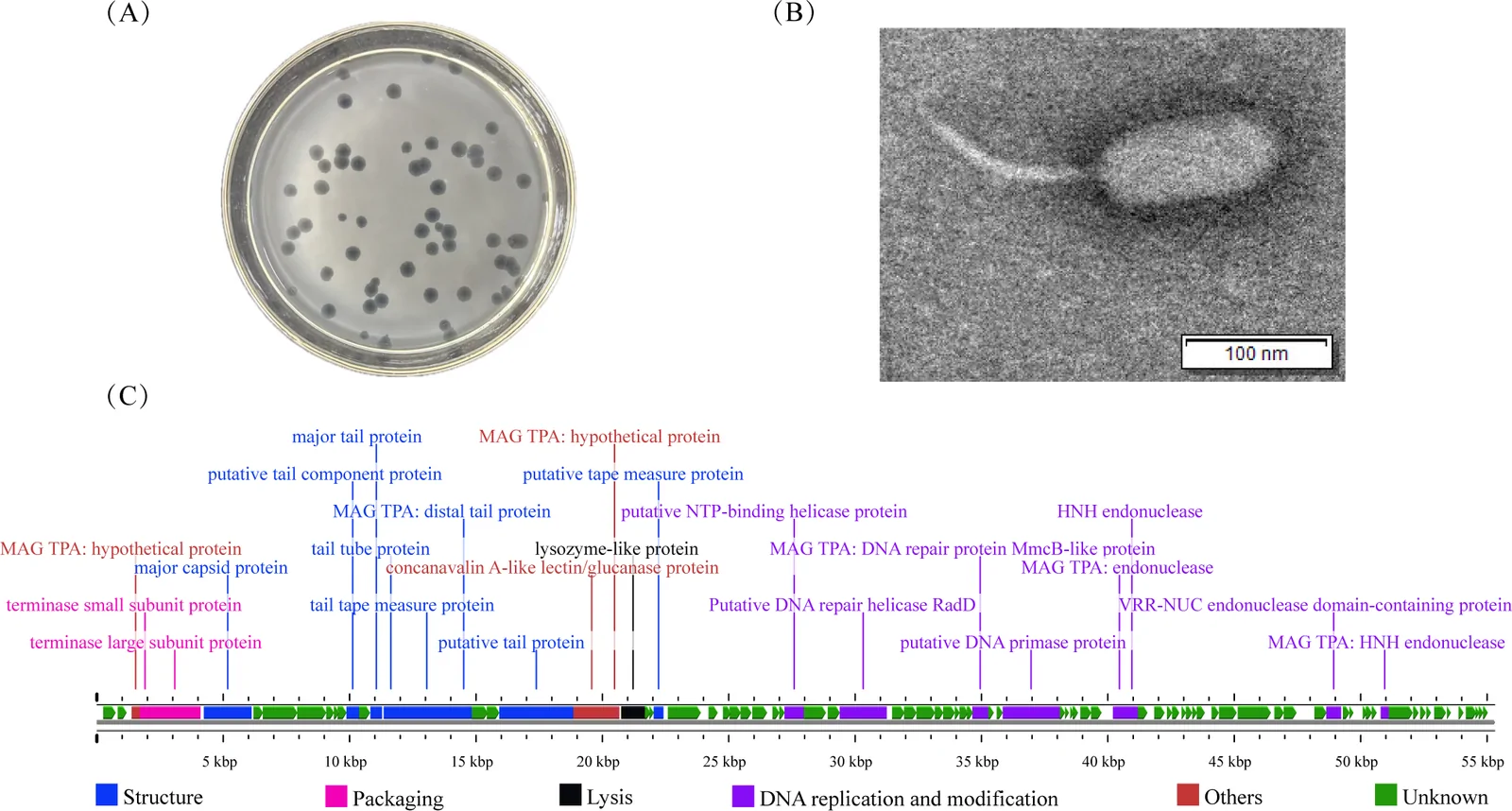
Morphology and genomic features of phage vB_AgrS_573. (**A**) Plaques of phage vB_AgrS_573 formed on a lawn of *A. tumefaciens* GV3101. (**B**) Transmission electron micrograph of phage vB_AgrS_573. Scale bar = 100 nm; magnification, ×150,000. (**C**) Genomic map of vB_AgrS_573 generated using the Proksee server.

Phage vB_AgrS_573 is a lytic phage with an icosahedral head and a long non-contractile tail (siphovirus morphology), exhibiting typical morphological features of the class *Caudoviricetes* (Fig. 1A and B). Its genome comprises a 55,316-bp linear double-stranded DNA with 52.19% GC content, encoding 91 open reading frames (ORFs); 22 ORFs are functionally annotated, and no transfer RNA genes are identified (Fig. 1C). No genes related to bacterial virulence or antimicrobial resistance are detected. NCBI BLASTn analysis shows that vB_AgrS_573 shares the highest nucleotide sequence similarity (74.56%) with *Rhizobium* phage 16-3 (GenBank accession no. NC_011103). This newly identified phage genome expands the available repository of lytic *Agrobacterium* phages, offering valuable resources for the development of phage-based biocontrol against *Agrobacterium*.

## Data Availability

The near-complete genome sequence and associated data for phage vB_AgrS_573 have been deposited in GenBank under accession number PZ266826.1. The associated BioProject, Sequence Read Archive, and BioSample numbers are PRJNA1432219, SRR37634568, and SAMN56341712, respectively.
